# The Story of the Insane from Year to Year

**Published:** 1893-09-23

**Authors:** 


### THE STORY OF THE INSANE FROM YEAR TO YEAR.

Sixth Series.

### THE COPPICE LUNATIC HOSPITAL, NOTTINGHAM.

On January 1st, 1892, there were 94 patients in residence, and at the end of the year the numbers had fallen very slightly. The total cases under treatment during the year reached the number of 115; the admissions were 21, the recoveries 4, and the deaths 9. In addition to those discharged recovered we notice that 6 were discharged relieved. In dealing with such small numbers percentages of recoveries and deaths do not mean much and need not be quoted. Of the admissions 6 were due to hereditary influence and 2 to drink. A most instructive table is given at page 25. It shows the weekly payments of all the patients in the asylum during the year 1892. We learn that 50 paid £2 a week, 24 paid 30s., 19 paid sums varying from 15 to 30s., and in 3 the payments varied from 10s. to 15s. Considering the size of the hospital the number of cases received at low payments must be looked on as fairly satisfactory, and with another fifty beds it could no doubt be largely increased. It is much to be desired that the governors of other lunatic hospitals would strictly limit their highest rate to something like £2 a week. Their first duty is to relieve the needy. The Commissioners in Lunacy say the "house is in excellent order," and the Committee "wish to record their entire satisfaction with the manner in which Dr. Tate has discharged his onerous and responsible duties."

### WILTS COUNTY ASYLUM, DEVIZES.

There were resident on January 1st, 1892, 682 patients, and at the close of the year the numbers had risen to 693. The admissions were 121, the recoveries 51, and the deaths 55. Drink is given as the assigned cause of insanity in 10 of the admissions, and hereditary influence in 16. The table showing the various causes has been very carefully prepared, for in only one instance is the cause of the disease returned as unascertained. Twenty-five of the deaths were due to some form of brain mischief and 6 to consumption. It is always a pleasure to turn over the leaves of Mr. Bowes' report which contains everything it should contain and nothing that it should not. There are a few sensible remarks on post-mortem examinations, but we think if Mr. Bowes can keep up the average of 39 examinations out of 55 deaths he has approached the limit at which these examinations can be at all necessary in asylums. The visitors say they "have the pleasure of testifying to the careful and efficient management of the asylum by the Medical Superintendent, and they have frequent evidence of the goodwill borne towards him from the letters of discharged patients." The weekly rate of maintenance is 8s. 9d.

